# Self-compassion, psychological distress, and mental well-being in a predominantly young student sample: Cross-sectional associations and continuous interaction analysis

**DOI:** 10.1371/journal.pmen.0000653

**Published:** 2026-10-15

**Authors:** Ying Shi, Zhihui Cheng

**Affiliations:** 1 Department of Psychological Sciences and Health, University of Strathclyde, Glasgow, Scotland, United Kingdom; 2 STREAM Institute of Law and Psychology, Hong Kong, China; PLOS: Public Library of Science, UNITED KINGDOM OF GREAT BRITAIN AND NORTHERN IRELAND

## Abstract

This study examined the cross-sectional associations of self-compassion and psychological distress with mental well-being in a predominantly young student sample and tested whether self-compassion modifies the association between distress and well-being. The dual-continua framework guided the simultaneous consideration of distress and positive mental health. Participants (N = 278) completed the Self-Compassion Scale–Short Form (SCS-SF), General Health Questionnaire-12 (GHQ-12), and Warwick–Edinburgh Mental Well-Being Scale (WEMWBS). The principal analysis used mean-centred continuous predictors in hierarchical multiple regression, followed by simple-slopes and Johnson–Neyman analyses. Heteroscedasticity-consistent standard errors and sensitivity analyses were used to examine robustness. Secondary analyses comprised a median-split ANOVA examining categorical main and interaction effects and stratified Spearman correlations estimating the self-compassion–well-being association within distress groups. Higher self-compassion was associated with greater well-being, B = 0.230, 95% CI [0.126, 0.334], p < .001, whereas greater distress was associated with lower well-being, B = -1.450, 95% CI [-1.685, -1.214], p < .001. The continuous interaction was small, B = -0.028, 95% CI [-0.055, -0.001], ΔR² = .008, p = .039. The negative coefficient indicated that the positive self-compassion–well-being association weakened as distress increased and ran counter to the hypothesised protective buffering effect. The interaction was also sensitive to influential-case exclusions, alternative GHQ-12 scoring, and bootstrap estimation, while the secondary median-split ANOVA interaction was non-significant, p = .875. Higher self-compassion and lower psychological distress were independently associated with greater well-being. The small, specification-sensitive interaction provided no evidence of the hypothesised protective buffering effect. Parallel assessment of distress and well-being is warranted, while causal, clinical, and structural conclusions require longitudinal, experimental, and latent-variable research.

## Introduction

### Self-compassion and the dual continua model

Self-compassion comprises self-kindness, common humanity, and mindfulness [1]. Together these facets foster a caring stance toward difficulty [2], situate suffering within shared human experience [3], and cultivate non-judgmental awareness that helps curb rumination and avoidance [4]. Empirically, self-compassion has been linked to emotion regulation, stress reduction, and coping capacity [5], and to psychological well-being among adolescents [6], while gratitude partly accounts for its association with well-being [7]. Aggregate self-compassion scores may conceal variation among their constituent processes. Pandey et al. found that positive self-compassion mediated the relationships of self-esteem with hedonic and eudaimonic well-being, while negative self-compassion showed no corresponding indirect effect [8]. A later study identified distinct and oppositely directed indirect associations of positive and negative self-compassion with self-esteem and self-forgiveness [9]. As correlational evidence, these findings indicate that the components combined within an overall self-compassion score may relate differently to positive functioning. Overall self-compassion has also been positively associated with interdependent happiness among married Hindu couples, although this evidence was derived from a culturally specific correlational study [10].

The dual-continua model conceptualises mental well-being and psychological distress as correlated but distinguishable dimensions [11,12]. Related adolescent evidence indicates that stress, psychological distress, and well-being are closely associated but not interchangeable, with some relationships varying by gender [13]. Earlier findings illustrate this distinction: project mastery and strain were associated with variation in both distress and well-being, whereas project support was more specifically related to well-being and project hindrance to both outcomes [14]. Also, among women with chronic musculoskeletal pain, well-being and low psychological distress only partially overlapped and showed different predictors [15]. As such, positive functioning and psychological symptoms should be measured in parallel, whereas latent dimensionality requires direct psychometric testing.

Studies of college students, intervention participants, and adults with perfectionistic tendencies similarly associate self-compassion with subjective well-being, although the strength and pathways of these associations vary across samples [16–18]. Meta-analytic evidence associates self-compassion with lower depression [19], while positive and negative self-compassion components show different relationships with well-being and distress, with some variation according to cultural dialecticism [20]. Studies of people living with illness and athletes similarly indicate variation according to health conditions, performance demands, and social context [21,22]. The association between self-compassion and well-being may consequently depend on both the components measured and the context in which they operate.

### Self-compassion and well-being

Across clinical and non-clinical contexts, studies of people living with illness and athletes associate self-compassion with lower distress and better adjustment, although the strength of these relationships varies [21,22]. Intervention research also reports improvements in psychosocial outcomes following self-compassion interventions [23]. Broader positive-psychology interventions have improved well-being and reduced distress in clinical populations [24], while associations between self-compassion and distress vary in the contexts of childhood maltreatment and perceived discrimination [25,26]. A meta-analysis reported a moderately strong association between self-compassion and well-being (r = .47), although much of the evidence was drawn from North American samples [27]. Whether this association remains equally strong under elevated distress remains uncertain, creating a distinction between a general positive association and a protective buffering effect.

Systematic reviews and controlled trials indicate that self-compassion and compassion-focused interventions may improve self-compassion and several mental-health outcomes [23,28,29]. A related trial protocol examines compassion-focused therapy for reducing self-stigma among people with mental disorders [30], while a narrative review reports generally favourable, although methodologically varied, findings for positive mental-health outcomes [31]. Whether self-compassion remains associated with well-being after distress is controlled and whether this association varies across distress levels remain uncertain.

### Distress and well-being

Across the literature, psychological distress generally co-varies with lower well-being, yet the association is not strictly inverse. Dual-factor accounts conceptualise well-being and distress as related but distinguishable dimensions, allowing positive functioning and psychological symptoms to vary partly independently [11,12,32,33]. Within this framework, the availability of psychological and contextual resources appears to shape the strength of the association. Mindfulness and social support may contribute to differences in well-being among individuals reporting similar levels of distress [14,34]. Structural independence between distress and well-being, however, requires direct latent-variable testing.

Adaptive coping and positive approach are associated with positive well-being, whereas stress and neuroticism are more closely related to negative well-being [35]. Mindfulness facets have been linked to lower emotional distress partly through psychological well-being [34]. Self-compassion is inversely associated with distress in adolescents and shows context-specific associations among athletes, older adults, and people with Parkinson’s disease [22,36–38]. Self-compassion and emotion regulation have also been associated with psychological distress among university students [39]. Across structural and predictor-based studies, positive and negative mental-health indicators show both shared and specific correlates [32–35]. The self-compassion–well-being association may therefore include general and context-dependent components, requiring the direction of any interaction to be specified theoretically.

### Conditional associations between self-compassion, distress, and well-being

From a dual-continua perspective, self-compassion may be associated with higher well-being and lower distress through partly distinct associations [11,19,27]. Protective buffering places a more specific requirement on this pattern: higher self-compassion should attenuate the inverse distress–well-being association. Therefore, the direction of the interaction distinguishes a general positive correlate from a conditional protective resource.

Median splits reduce variability and statistical power and may obscure or distort interaction effects [40–42]. Measurement choices can further compress the available variation; the GHQ-12’s bimodal scoring may be sensitive to item phrasing and provide a restricted score range in non-clinical samples [43]. Continuous interaction modelling preserved the full variability of both predictors and served as the principal analysis; the median-split ANOVA was retained as a secondary analysis to examine the consequences of dichotomisation.

### Research questions and hypotheses

Previous research consistently associates self-compassion with positive functioning [5,7,27], while evidence for conditional protection under elevated distress remains context-specific, including perceived discrimination and illness-related threat [21,26]. The dual-continua framework conceptualises distress and well-being as correlated dimensions that warrant parallel assessment [11,12,44]. The current cross-sectional analysis examines observed associations and conditional relationships in a predominantly young, student-based sample. Direct tests of latent structure and causal processes require different research designs.

Three research questions corresponded to the principal hypotheses: whether self-compassion was positively associated with well-being (RQ1), whether distress was negatively associated with well-being (RQ2), and whether self-compassion altered the distress–well-being association in the direction predicted by protective buffering (RQ3). RQ4 examined descriptively whether the positive self-compassion–well-being association was observable within both lower- and higher-distress strata.

Self-compassion is positively associated with well-being and positive psychological functioning [5,7,27]. The dual-continua framework conceptualises well-being and psychological distress as related but distinguishable dimensions [11,12,44], while research in chronic pain and other populations reports partially distinct correlates of these outcomes [14,15]. Therefore, H1 predicted that higher self-compassion would be associated with higher mental well-being, and H2 predicted that higher psychological distress would be associated with lower mental well-being. Evidence of context-dependent associations across illness and athletic settings [21,22], together with longitudinal evidence of buffering under perceived discrimination [26], motivated H3: self-compassion was expected to buffer the inverse distress–well-being association, requiring a positive self-compassion × distress coefficient under the present scoring directions.

H4 concerned the consistency of the self-compassion–well-being association across distress levels. Meta-analytic and empirical evidence generally indicates a positive association between self-compassion and well-being [5,7,27], while studies of adolescents, athletes, people living with illness, older adults, and clinical samples suggest that the strength and expression of this association may vary across contexts [21,22,36–38,45,46]. H4 predicted positive correlations between self-compassion and well-being in the full sample and within both lower- and higher-distress strata. It predicted the presence of an association within each stratum, not a difference between the subgroup correlations.

## Method

### Participants

The study targeted a non-clinical sample aged 16 years or older who could read and understand English. In accordance with the original eligibility criteria, individuals self-reporting a clinically diagnosed mental health condition, such as depression or anxiety, were excluded. This criterion reflected the intended non-clinical sampling frame, restricted the observed range of distress, and limited application of the findings to people with diagnosed conditions.

Participants were recruited via multiple channels to maximise accessibility: the University of Glasgow SONA system (enabling Level 1 psychology students to receive course credit), the School’s Participant Recruitment channel on Microsoft Teams and its Experiment Pool, and broader outreach through social media and personal/professional networks.

Based on effect sizes reported in related reviews [47,48], the original a priori power analyses covered the initial categorical and correlational analyses. Assuming a medium effect (*f* = .25), α = .05, and 80% power, a 2 × 2 ANOVA required *N* = 128, or approximately 32 participants per cell. For the stratified correlations, at least 88 participants per distress group were required to detect *r* = .30 with 80% power. Allowing for exclusions, the recruitment target exceeded *N* = 176. The continuous interaction model was subsequently prioritised and was interpreted through its effect size, confidence interval, and sensitivity analyses because it was outside the original power calculation.

In total, 308 individuals opened the survey, of whom 278 were retained in the analytic dataset. The remaining 30 records were excluded because they comprised 2 non-consent cases, 8 non-completed surveys, and 20 cases with missing item-level data for one or more target scales. All 278 retained participants had complete item-level data for the three target scales and were included in the analyses. The analytic sample was predominantly young and student-based, and most participants identified as women.

Data collection took place from November 2024 to February 2025. All participants provided informed consent electronically. For participants aged 16–17 years, electronic informed consent was obtained directly from the participants, as approved by the ethics committee; parental or guardian consent was not required under the approved protocol. Ethical approval was granted by the University of Glasgow, College of Medical, Veterinary and Life Sciences Research Ethics Committee (Application No. 200240079).

### Materials

The online questionnaire included demographic items (age, gender identity, ethnicity, student status) and three validated scales. Age was recorded via a dropdown. Gender identity offered options (“Man,” “Woman,” “Non-binary,” “Other,” “Prefer not to say”) with an open text field to support inclusive self-description [49]. Ethnicity was captured with an open response to allow detailed self-report. Student status was recorded as Yes/No.

The Self-Compassion Scale – Short Form (SCS-SF) [50,51] comprises 12 items rated 1–5 (“almost never” to “almost always”) tapping self-kindness, self-judgment, common humanity, isolation, mindfulness, and over-identification. Following standard practice, six negatively worded items were reverse-scored prior to summing. Total scores range 12–60, with higher scores indicating greater self-compassion. Prior work supports strong psychometric properties, including internal consistency and convergence with the long form [51]. An example item is: *“When I fail at something important to me, I become consumed by feelings of inadequacy.”*

The General Health Questionnaire-12 (GHQ-12) [52] indexes recent psychological distress with 12 items (six positively and six negatively keyed). All GHQ-12 items were scored using the conventional 0–0–1–1 bimodal method in the direction of increasing psychological distress, producing a total score from 0 to 12. The GHQ-12 is widely validated [53] and shows satisfactory reliability across settings, including student and non-student samples [54,55]. Example item: *“Have you recently felt you were playing a useful part in things?”*

The Warwick-Edinburgh Mental Well-Being Scale (WEMWBS) [56] contains 14 positively worded items rated 1–5 (“none of the time” to “all of the time”). Total scores range 14–70; higher scores indicate better mental well-being. Evidence supports high internal consistency across populations [57,58]. Example item: *“I’ve been feeling optimistic about the future.”* No new scale items were developed, and internal consistency in the present sample was evaluated using Cronbach’s α and McDonald’s ω; the coefficients are reported in the Results.

### Procedure

The study was delivered via Qualtrics. Invitations included a secure survey link. After reading the Participant Information Sheet, individuals indicated consent electronically. Eligibility screening preceded survey items; those ineligible or declining consent could not proceed.

The survey presented demographic questions first, followed by the SCS-SF, GHQ-12, and WEMWBS in a fixed order. Only the age item was mandatory to enforce the age criterion. Upon submission, participants were reminded that, due to anonymity, individual data could not be withdrawn post-submission. A debrief page reiterated the study aims, provided contact details for the researcher, and listed mental health support resources. Completion time was approximately 10–15 minutes.

### Design and data analysis

The study used a cross-sectional design. Analyses were conducted in R version 4.4.2 using RStudio 2024.12.0 + 467 [59,60]. Continuous multiple regression served as the principal analysis because it preserved the full variability of self-compassion and distress. Secondary analyses comprised a median-split ANOVA examining categorical main and interaction effects and stratified Spearman correlations estimating the self-compassion–well-being association within distress groups.

For the secondary 2 × 2 ANOVA, participants were classified using the sample medians. SCS-SF scores below 32 were assigned to the lower self-compassion group and scores of 32 or above to the higher group. GHQ-12 scores below 5 were assigned to the lower-distress group and scores of 5 or above to the higher-distress group. Values equal to the median were assigned to the higher group. Type III sums of squares were used because cell sizes were unequal. Despite the recognised loss of information, reduced power, and potential misclassification associated with dichotomisation [40–42], the median-split ANOVA provided secondary categorical tests of the H1 and H2 main associations and the H3 interaction; the continuous model provided the principal interaction test.

Spearman correlations were calculated between SCS-SF and WEMWBS scores in the full sample and descriptively within the lower- and higher-distress groups. Spearman’s ρ was used because the relationships were monotonic without an assumption of strict linearity. These analyses addressed H4 by estimating the positive association within each stratum. Numerical differences between the subgroup coefficients were interpreted descriptively because the analysis included no formal test of their difference.

For the principal analysis, SCS-SF and GHQ-12 scores were mean-centred. WEMWBS was first regressed on the two main effects, after which the self-compassion × distress product term was entered hierarchically. The change in R² associated with the interaction was reported alongside the full-model R² and adjusted R². Unstandardised coefficients (B), standard errors, 95% confidence intervals, standardised coefficients (β) with 95% confidence intervals, and p-values were reported. A buffering effect consistent with H3 required a positive interaction coefficient because higher GHQ-12 scores represented greater distress and higher WEMWBS scores represented better well-being.

The interaction was examined using simple slopes of self-compassion at the mean and at one standard deviation below and above the mean of distress. Conditional distress slopes were also estimated at lower, mean, and higher self-compassion. Johnson–Neyman analysis identified the observed distress values at which the conditional self-compassion slope changed statistical significance. An interaction plot based on continuous fitted values displayed the form and magnitude of the interaction.

For ANOVA, residual normality and homogeneity of variance were evaluated using residual diagnostics and Levene’s test. For regression, residual normality, heteroscedasticity, functional form, and multicollinearity were assessed using the Shapiro–Wilk test, Breusch–Pagan test, Ramsey RESET test, residual plots, and variance inflation factors. Independence was supported by the survey design and screening for duplicate participant identifiers. Potentially influential observations were identified using Cook’s distance, leverage, studentised residuals, and DFBETAs. These observations were retained in the principal analysis because they represented plausible responses, with case-exclusion models reported as sensitivity analyses.

Robustness across alternative specifications was examined using HC3 heteroscedasticity-consistent standard errors, GHQ-12 Likert scoring, quadratic terms for self-compassion and distress, adjustment for age and gender, and 5,000 bootstrap resamples with percentile and bias-corrected and accelerated confidence intervals.

SCS-SF scores were summed after reverse-scoring the six negatively worded items, producing a possible range of 12–60. GHQ-12 items were scored using the bimodal 0–0–1–1 convention for the principal analysis, producing a range of 0–12; 0–1–2–3 Likert scoring was examined in sensitivity analysis. WEMWBS items were summed to produce a possible range of 14–70. Complete-case analysis was specified for the three target scales, and all 278 analytic cases had complete data.

## Results

### Sample characteristics and analytic data

Of the 308 individuals who opened the survey, 278 were retained in the final analytic sample after the stated eligibility and completeness procedures. This represented 90.3% of those who opened the survey. The supplied analytic dataset contained no missing item responses on the SCS-SF, GHQ-12 or WEMWBS, and all 278 cases were therefore included in the analyses.

Table 1 summarises the demographic characteristics of the analytic sample. Participants had a mean age of 19.30 years (SD = 4.54), and 41 participants (14.75%) were aged 16 or 17 years. The sample was predominantly composed of students (98.20%) and women (83.09%).

**Table 1:** Demographic characteristics of the analytic sample (N = 278).

Demographic factor	Category	Frequency	Percent (%)	Mean	SD
Gender	Men	34	12.23		
	Women	231	83.09		
	Non-binary	13	4.68		
	Total	278	100		
Age	16–17 years	41	14.75	19.3	4.54
	18 years	131	47.12		
	19–25 years	93	33.45		
	>25 years	13	4.68		
	Total	278	100		
Student status	Yes	273	98.2		
	No	5	1.8		
	Total	278	100		

### Descriptive statistics and reliability

Descriptive statistics and sample-specific reliability estimates are presented in Table 2. SCS-SF scores ranged from 15 to 58 (M = 32.37, SD = 7.37, Mdn = 32.00, IQR = 28–37). GHQ-12 scores ranged from 0 to 12 (M = 5.31, SD = 3.21, Mdn = 5.00, IQR = 3–8), while WEMWBS scores ranged from 23 to 70 (M = 42.48, SD = 7.96, Mdn = 42.00, IQR = 37–48).

**Table 2:** Descriptive statistics and internal consistency estimates.

Measure	Observed range	Mean	SD	Median	IQR	α/KR-20 [95% CI]	ω
SCS-SF	15–58	32.37	7.37	32	28–37	.816 [.774,.848]	0.82
GHQ-12	0–12	5.31	3.21	5	3–8	.802 [.763,.833]	0.805
WEMWBS	23–70	42.48	7.96	42	37–48	.866 [.836,.890]	0.871

Note. α = Cronbach’s alpha; KR-20 = Kuder–Richardson coefficient for dichotomously scored items; ω = McDonald’s omega. Confidence intervals for α/KR-20 were estimated using 5,000 bootstrap samples.

All three measures demonstrated satisfactory internal consistency in the present sample. Cronbach’s α was.816 for the SCS-SF and.866 for the WEMWBS. For the dichotomously scored GHQ-12, the equivalent KR-20 estimate was.802. McDonald’s ω estimates were similar, ranging from.805 to.871.

### Continuous regression analysis

In the principal continuous regression, self-compassion and distress jointly accounted for 51.04% of the variance in mental well-being, F(2, 275) = 143.40, p < .001, adjusted R² = .507. Higher self-compassion was associated with higher well-being, B = .249, SE = .052, 95% CI [.147,.352], p < .001, while higher distress was associated with lower well-being, B = −1.421, SE = .120, 95% CI [−1.657, −1.185], p < .001. These results supported H1 and H2.

Adding the self-compassion × distress term produced a statistically significant model, F(3, 274) = 98.15, p < .001, R² = .518, adjusted R² = .513. Self-compassion remained positively associated with well-being, B = .230, SE = .053, 95% CI [.126,.334], standardised β = .213, p < .001, and distress remained negatively associated with well-being, B = −1.450, SE = .120, 95% CI [−1.685, −1.214], standardised β = −.585, p < .001.

The interaction coefficient was negative and conventionally significant, B = −.028, SE = .014, 95% CI [−.055, − .001], standardised β = −.083, p = .039. The HC3-robust estimate was similar, SE = .014, 95% CI [−.055, − .001], p = .043. However, the interaction increased explained variance by only ΔR² = .0076, F-change(1, 274) = 4.30, p = .039, corresponding to f² = .016 and partial R² = .015. Its incremental contribution was therefore small (Table 3).

**Table 3:** Hierarchical regression predicting mental well-being (N = 278).

Predictor	Model 1 B (SE)	95% CI	p	Model 2 B (SE)	95% CI	Standardised β [95% CI]	p
Intercept	42.482 (.335)	[41.822, 43.142]	<.001	42.160 (.368)	[41.437, 42.884]	—	<.001
Self-compassion	.249 (.052)	[.147, .352]	<.001	.230 (.053)	[.126,.334]	0.213 [.117,.309]	<.001
Distress	−1.421 (.120)	[−1.657, −1.185]	<.001	−1.450 (.120)	[−1.685, −1.214]	−.585 [−.680, −.489]	<.001
Self-compassion × distress	—	—	—	−.028 (.014)	[−.055, −.001]	−.083 [−.163, −.004]	0.039
R²	0.510			0.518			
Adjusted R²	0.507			0.513			
ΔR²				0.008			0.039

Note. B denotes the unstandardised coefficient. Model 1 included the two continuous main effects. Model 2 added the interaction term. Predictors were mean-centred before estimation. HC3-robust inference for the interaction was p = .043. Standardised coefficients and their confidence intervals were estimated from the model fitted to z-standardised variables.

### Conditional associations

The negative interaction indicated that the positive association between self-compassion and well-being became weaker as distress increased. At one standard deviation below the mean of distress (raw GHQ-12 = 2.10), self-compassion was positively associated with well-being, B = .320, SE = .062, 95% CI [.198,.443], p < .001. The association remained positive at mean distress (GHQ-12 = 5.31), B = .230, SE = .053, 95% CI [.126,.334], p < .001. At one standard deviation above the mean (GHQ-12 = 8.52), the slope was smaller and did not reach conventional significance, B = .140, SE = .074, 95% CI [−.006,.286], p = .059. HC3 inference produced the same pattern, with p = .060 for the high-distress slope.

Johnson–Neyman analysis identified a raw GHQ-12 score of approximately 8.40 as the point at which the self-compassion slope ceased to be statistically distinguishable from zero. Within the observed range, the self-compassion–well-being association was significant below this threshold and non-significant from approximately 8.40 to 12.00 (Fig 1).

**Fig 1:**
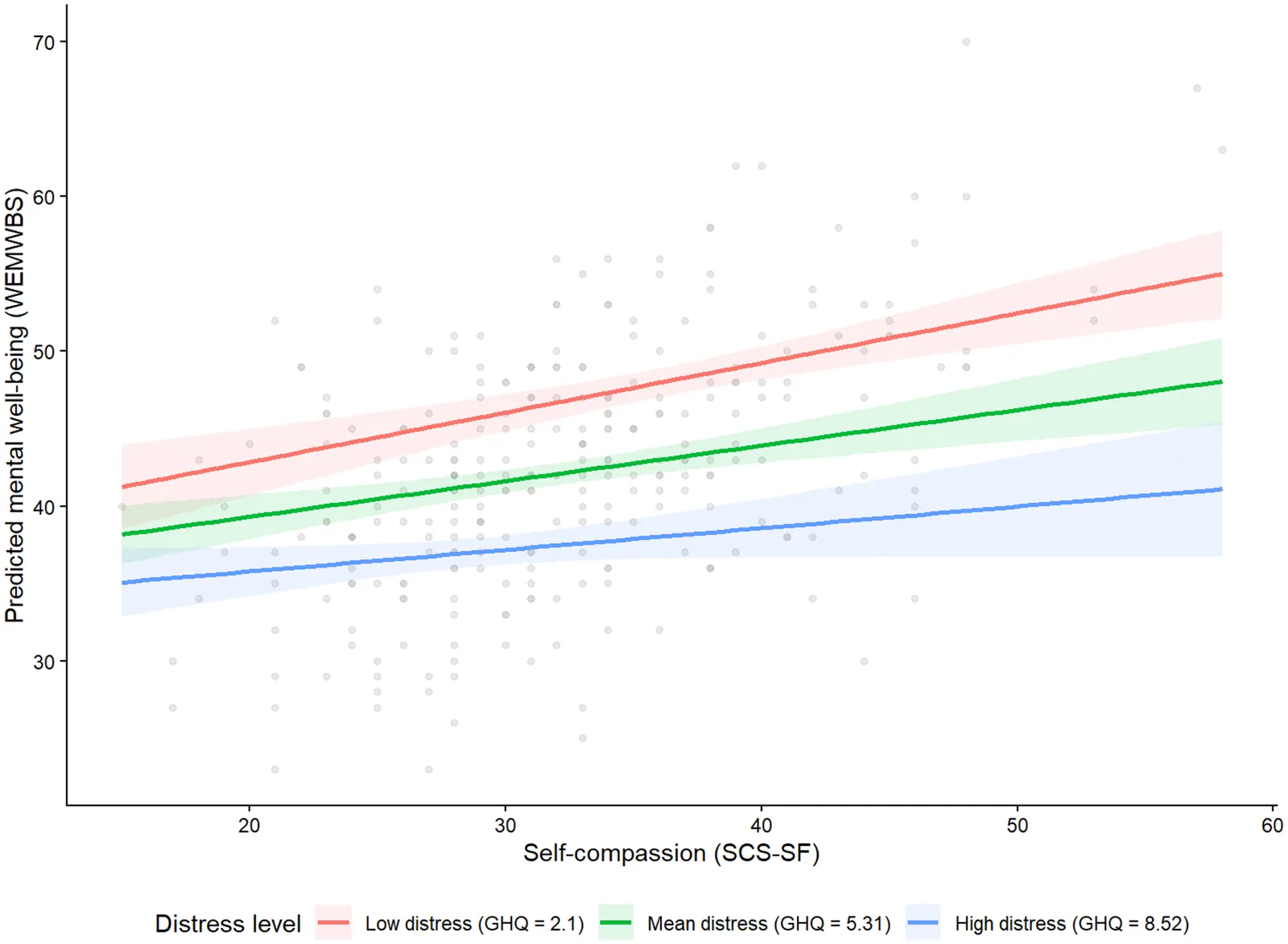
Conditional association between self-compassion and predicted mental well-being at low, mean and high distress. Note. Lines show predicted WEMWBS scores across SCS-SF scores at GHQ-12 values corresponding to one standard deviation below the mean, the mean and one standard deviation above the mean. Shaded areas represent 95% confidence intervals; grey points show observed values.

Probing the interaction in the reverse direction did not produce the hypothesised buffering pattern. Distress was negatively associated with well-being at low, mean and high self-compassion. Moreover, the estimated negative slope became larger as self-compassion increased: B = −1.243 at low self-compassion, B = −1.450 at the mean and B = −1.656 at high self-compassion, all ps < .001 (Table 4).

**Table 4:** Conditional slopes from the continuous interaction model.

Focal association	Moderator level and raw score	B	SE	95% CI	t	p
Self-compassion → well-being	Low distress, GHQ = 2.10	0.32	0.062	[.198,.443]	5.16	<.001
Self-compassion → well-being	Mean distress, GHQ = 5.31	0.23	0.053	[.126,.334]	4.37	<.001
Self-compassion → well-being	High distress, GHQ = 8.52	0.14	0.074	[−.006,.286]	1.89	0.059
Distress → well-being	Low self-compassion, SCS-SF = 25.01	−1.243	0.147	[−1.532, −.953]	−8.46	<.001
Distress → well-being	Mean self-compassion, SCS-SF = 32.37	−1.450	0.12	[−1.685, −1.214]	−12.09	<.001
Distress → well-being	High self-compassion, SCS-SF = 39.74	−1.656	0.165	[−1.980, −1.333]	−10.07	<.001

Note. Low and high moderator values represent one standard deviation below and above the sample mean. The table reports conventional standard errors; HC3 estimates led to the same substantive conclusions.

### Regression diagnostics and sensitivity analyses

The primary model showed no clear evidence of residual non-normality, W = .993, p = .270, or heteroscedasticity, Breusch–Pagan χ²(3) = 1.83, p = .609. The Ramsey RESET test was non-significant, F = 2.73, p = .067; nonlinear specifications were subsequently examined in sensitivity analyses. Multicollinearity was low, with VIFs ranging from 1.03 to 1.35. Influence diagnostics identified 13 observations exceeding Cook’s D > 4/N, 25 exceeding the leverage criterion, one with an absolute studentised residual above 3 and 37 exceeding at least one DFBETA threshold. These cases were retained in the primary analysis; case-deletion models were used only to assess sensitivity.

As Table 5 shows, the negative interaction remained conventionally significant after adjustment for age and gender, B = −.028, p = .040, HC3 p = .046. It became non-significant when GHQ-12 was scored using the Likert method, B = −.012, p = .064, HC3 p = .079, and after quadratic terms were introduced, B = −.012, p = .545. The interaction also became non-significant after excluding observations exceeding the Cook’s distance or leverage thresholds. Removing the single case with an absolute studentised residual above 3 produced p = .044 under conventional inference but HC3 p = .050.

**Table 5:** Sensitivity analyses for the self-compassion × distress interaction.

Specification	N	B	SE	95% CI	p	HC3 p
Primary bimodal model	278	−.0281	0.0135	[−.0547, −.0014]	0.039	0.043
Adjusted for age and gender	278	−.0281	0.0136	[−.0550, −.0013]	0.04	0.046
GHQ-12 Likert scoring	278	−.0120	0.0064	[−.0246,.0007]	0.064	0.079
Quadratic terms included	278	−.0119	0.0196	[−.0504,.0267]	0.545	0.544
Cook’s D > 4/N cases excluded	265	−.0200	0.0145	[−.0485,.0085]	0.169	0.087
High-leverage cases excluded	253	−.0021	0.0237	[−.0488,.0446]	0.929	0.929
One case with |studentised residual| > 3 excluded	277	−.0270	0.0133	[−.0533, −.0007]	0.044	0.05

The 5,000-replication bootstrap produced mixed confidence-interval evidence. The percentile interval included zero, 95% CI [−.0529,.0002], while the bias-corrected and accelerated interval only narrowly excluded zero, 95% BCa CI [−.0537, − .0004]. Considered together with the alternative GHQ scoring, nonlinear model and influence-case analyses, the interaction was small and sensitive to analytic specification. The principal continuous model therefore identified an exploratory conditional pattern, but H3 was not robustly supported.

### Median-Split ANOVA

The median-split ANOVA was treated as a secondary categorical analysis. Scores equal to the sample medians were assigned to the high groups, producing cut-offs of 32 for SCS-SF and 5 for GHQ-12. Cell sizes were unequal: low self-compassion/low distress, n = 34; high self-compassion/low distress, n = 87; low self-compassion/high distress, n = 103; and high self-compassion/high distress, n = 54. Type III sums of squares were therefore used. Residual normality, W = .995, p = .492, and homogeneity of variance, F(3, 274) = 1.55, p = .201, showed no clear assumption violations. Independence was supported by the presence of one unique response ID for each participant.

The Type III ANOVA detected a main association of self-compassion with well-being, F(1, 274) = 22.15, p < .001, partial η² = .075, supporting H1 in the secondary categorical analysis. Participants in the high-self-compassion group reported higher well-being (M = 45.78, SD = 7.76) than those in the low-self-compassion group (M = 39.09, SD = 6.63). Distress also showed a significant main association, F(1, 274) = 75.82, p < .001, partial η² = .217. The high-distress group reported lower well-being (M = 38.64, SD = 6.60) than the low-distress group (M = 47.46, SD = 6.72), supporting H2.

The categorical self-compassion × distress interaction was negligible and non-significant, F(1, 274) = 0.02, p = .875, partial η² < .001. At low distress, mean well-being was 48.54 (SD = 7.15) in the high-self-compassion group and 44.71 (SD = 4.48) in the low-self-compassion group. At high distress, the corresponding means were 41.33 (SD = 6.60) and 37.23 (SD = 6.18). The near-parallel group differences provided no categorical evidence for H3 (Table 6).

**Table 6:** Type III two-way ANOVA predicting mental well-being.

Effect	F	df	p	Partial η²	One-sided 95% CI
Self-compassion group	22.15	1, 274	<.001	0.075	[.032, 1.000]
Distress group	75.82	1, 274	<.001	0.217	[.150, 1.000]
Self-compassion × distress	0.02	1, 274	0.875	<.001	[.000, 1.000]

Note. Effect-size confidence intervals are one-sided; the upper bound is fixed at 1.00. The analysis used sum contrasts and Type III sums of squares because cell sizes were unequal.

### Descriptive subgroup correlations

As shown in Table 7, self-compassion was positively associated with well-being in the full sample, ρ = .464, 95% CI [.364,.554], p < .001. Positive associations were also observed in the high-distress group, ρ = .306, 95% CI [.152,.445], p < .001, and the low-distress group, ρ = .347, 95% CI [.175,.499], p < .001. These findings supported the limited claim in H4 that the positive self-compassion–well-being association was observable within both distress strata.

**Table 7:** Spearman correlations between self-compassion and mental well-being.

Sample	N	ρ	95% CI	p
Full sample	278	0.464	[.364,.554]	<.001
High-distress group	157	0.306	[.152,.445]	<.001
Low-distress group	121	0.347	[.175,.499]	<.001

Note. Distress groups were defined using the sample median of 5, with scores equal to 5 assigned to the high-distress group.

Although the coefficient was numerically larger in the low-distress group, the subgroup correlations were not formally compared. They therefore provide no independent evidence that the association differed between distress groups and should not be interpreted as confirmation of H3.

## Discussion

### Summary of main findings

Higher self-compassion was consistently associated with greater mental well-being, supporting H1, whereas greater psychological distress was associated with lower well-being, supporting H2. These associations were observed in both categorical and continuous analyses. H3 was not supported. The continuous interaction between self-compassion and distress was statistically significant (*B* = −0.028, *p* = .039), but its negative direction indicated that the positive association between self-compassion and well-being weakened as distress increased. This pattern contradicted the hypothesised buffering effect. The interaction explained less than 1% of additional variance (Δ*R*² = .008) and was not consistently significant across sensitivity analyses. H4 was supported within its descriptive scope: self-compassion was positively correlated with well-being in the full sample and within both distress groups. However, the subgroup correlations were not formally compared and therefore provide no evidence of moderation.

### Positive association between self-compassion and well-being

Higher self-compassion was associated with greater well-being in the categorical analysis and remained positively associated with well-being after adjustment for distress in the continuous model. This finding accords with meta-analytic and broader theoretical evidence linking self-compassion to psychological well-being [27,61–63], quantitative research on positive self-compassion [8], and qualitative accounts describing self-compassion as a source of emotional balance and adaptive self-relating [64]. The cross-sectional design precludes causal conclusions, and the observed association may also reflect broader characteristics such as emotional regulation, reduced self-criticism, or lower rumination.

Self-compassion was positively correlated with well-being in both the low-distress and high-distress groups. The association was numerically stronger among participants with lower distress, but the subgroup coefficients were not formally compared. Their numerical ordering therefore cannot establish a difference between groups. The continuous interaction analysis provided a direct test of conditional association and indicated only a small, unstable attenuation of the self-compassion coefficient at higher levels of distress.

### Negative association between distress and well-being

Higher psychological distress was strongly associated with lower mental well-being, supporting H2. Self-compassion retained a positive association with well-being after distress was included in the regression model, indicating that the two variables contributed distinct statistical information. This pattern is consistent with the dual-continua position that psychological symptoms and positive mental health represent related but distinguishable dimensions [11,33]. Structural independence remains untested because the analysis did not include latent model comparisons, profile analysis, or longitudinal assessments of change across the two dimensions.

Project mastery, strain, and self-involvement have been associated with variation in both distress and well-being, while project support was more specifically related to well-being and project hindrance to both outcomes [14]. Among women with chronic musculoskeletal pain, well-being and low psychological distress also showed only partial overlap and had different predictors [15]. Common and outcome-specific predictors may therefore coexist [34]. The strong inverse association observed here, may reflect overlapping responses to adverse circumstances alongside distinct contributions from personal and contextual resources.

### Interaction between self-compassion and distress

The median-split analysis produced no significant interaction between self-compassion and distress. Dichotomising continuous variables reduces information and statistical power and may obscure or distort interaction effects [40–42]. The continuous regression model identified a statistically significant interaction, but the effect was small and inconsistent across alternative specifications. The estimated self-compassion coefficient decreased from *B* = .320 at lower distress to *B* = .140 at higher distress, with the latter simple slope narrowly missing conventional significance (*p* = .059). The observed pattern therefore indicated attenuation of the positive self-compassion–well-being association as distress increased. A buffering effect would require the negative association between distress and well-being to weaken at higher levels of self-compassion; that pattern was absent.

In sensitivity analyses, the interaction remained conventionally significant with HC3 standard errors and after adjustment for age and gender, but became non-significant under alternative GHQ-12 scoring, the quadratic specification, and the Cook’s-distance and leverage case-exclusion models. Its incremental explanatory contribution was also small (Δ*R*² = .008). The coefficient may reflect sampling variability, model dependence, or a weak conditional association requiring replication. The available evidence was insufficient to establish a robust interaction, and the hypothesised buffering effect was unsupported.

### Theoretical contributions

Self-compassion remained positively associated with well-being after adjustment for distress, whereas the hypothesised buffering effect received no robust support. These findings constrain claims that self-compassion necessarily protects well-being more strongly under elevated distress. They are also compatible with dual-continua models in which positive mental health and psychological symptoms are associated yet partly distinct [11,33], although direct examination of their structural independence requires latent-variable analysis.

The aggregate SCS-SF score may conceal differences among compassionate and uncompassionate forms of self-responding. Qualitative evidence has described self-compassion as involving emotional balance, reduced self-criticism, and greater tolerance of difficult experiences [64]. Quantitative studies have similarly reported distinct associations for positive self-compassion and uncompassionate self-responding [8,9,65]. The present interaction cannot be attributed to specific components because the total score combines these processes. Future research should examine whether self-kindness, common humanity, mindfulness, self-judgement, isolation, and over-identification show different associations with well-being across levels of distress.

Longitudinal models could distinguish moderation, mediation, and reciprocal association by examining whether self-compassion predicts subsequent changes in distress and well-being, whether distress alters later self-compassion, and whether these relationships vary across time or context. Latent-variable and person-centred analyses could also determine whether distinct combinations of distress and well-being correspond to different self-compassion profiles.

### Practical implications and future intervention research

No intervention was administered, and participants with diagnosed mental health conditions were excluded. Clinical inferences concerning the effectiveness, sequencing, or delivery of compassion-focused interventions are therefore premature. Existing intervention research suggests that compassion-based approaches can reduce psychological distress and improve well-being, although effects vary across populations and study designs [23,28,29,31]. Future intervention studies should assess self-compassion, distress, and well-being simultaneously and directly test whether adapting treatment to distress level improves outcomes.

Future trials could test whether participants experiencing substantial distress require additional support before or during compassion-focused work. Distress Brief Intervention provides one model of rapid, compassionate support for people experiencing distress [66]. Its optimal placement in relation to compassion-focused therapy, and any benefit of combining the approaches, require direct evaluation. Staged-care proposals should therefore be tested through preregistered trials with repeated measures, clearly defined adaptation rules, and clinically relevant outcomes.

### Limitations and future research directions

Most participants were students (98.2%), 83.09% identified as women, and the mean age was 19.3 years; 41 participants (14.75%) were aged 16–17 years. Recruitment through university channels, social media, and personal or professional networks may have introduced self-selection. Excluding participants who reported diagnosed mental health conditions may have restricted the range of distress and limited generalisability to clinical populations. Ethnicity was collected through open-text responses, which did not support detailed subgroup analysis. The culturally and demographically concentrated sample also limits generalisation beyond comparable student populations [67]. Future research should recruit community and clinical participants across wider age ranges and use clearly defined demographic categories that permit adequately powered subgroup comparisons.

All variables were measured using self-report questionnaires at a single time point, creating possible common-method variance and preventing conclusions about temporal ordering. Although the SCS-SF, GHQ-12, and WEMWBS showed acceptable internal consistency, measurement equivalence across demographic and distress groups was not examined. Future studies should assess measurement invariance before interpreting group differences or interaction effects [68]. Multi-method designs incorporating clinical interviews, behavioural indicators, or repeated assessments would provide stronger evidence.

The analysis plan was not preregistered. The initial ANOVA relied on median splits, and the continuous interaction model was subsequently prioritised because it retained the information contained in the original scores. The interaction finding should therefore be treated as requiring independent confirmation. Future studies should preregister continuous moderation models, specify covariates and sensitivity analyses in advance, report incremental effect sizes, and ensure adequate power for small interaction effects. Longitudinal and experimental designs are also needed to examine causal direction and changes in self-compassion, distress, and well-being over time.

## Conclusions

Higher self-compassion was consistently associated with greater mental well-being, whereas greater psychological distress was associated with lower well-being. Self-compassion also remained positively correlated with well-being within both distress strata. The continuous interaction was small, negative, and sensitive to alternative analytical specifications. Its direction indicated that the positive self-compassion–well-being association weakened as distress increased, providing no evidence of the hypothesised protective buffering effect. Therefore, distress and positive mental health should be assessed in parallel, although direct evaluation of the dual-continua model requires latent-variable analysis. The cross-sectional design, predominantly young student sample, exclusion of individuals with diagnosed mental health conditions, and reliance on self-report limit causal, clinical, and population-level inferences. Replication in more diverse samples using preregistered continuous analyses, longitudinal or experimental designs, and separate modelling of self-compassion components is required to clarify the observed attenuation pattern.

## Supporting information

S1 Fig Mean mental well-being by self-compassion and distress groups. Note. Points represent observed cell means and error bars represent ±1 standard error. The categorical interaction was not statistically significant.
(TIF)
